# Rectovaginal Fistula From Untreated Ulcerative Colitis in Pregnancy: A Case Report and Review of the Literature

**DOI:** 10.7759/cureus.40662

**Published:** 2023-06-19

**Authors:** Paulina C Altshuler, Eric Bradford, Annette van Swaay, Sarah Rabice, Rachel Gaffney

**Affiliations:** 1 Obstetrics and Gynecology, Intermountain Healthcare/Saint Joseph Hospital, Denver, USA; 2 General Surgery, Intermountain Healthcare/Saint Joseph Hospital, Denver, USA; 3 Female Pelvic Medicine and Reconstructive Surgery, University of Iowa, Iowa City, USA

**Keywords:** perianal disease, intrapartum care, inflammatory bowel disease, severe ulcerative colitis, ulcerative colitis, rectovaginal fistula

## Abstract

Inflammatory bowel disease can have reproductive consequences depending on disease severity at the time of conception and antepartum management. A 37-year-old G1 with ulcerative pancolitis initially did not disclose her medical history to the obstetrics providers. She developed worsening hematochezia and microcytic anemia and declined antepartum treatment of ulcerative colitis. She then developed a rectovaginal fistula, underwent cesarean delivery but declined intraoperative management of the fistula, and started treatment after significant postpartum weight loss. She was ultimately lost to follow-up care. For patients with ulcerative colitis, a multidisciplinary team approach should be utilized to identify barriers to care, prevent disease progression, and optimize pregnancy outcomes. Delivery methods should be individualized to the patient, and further studies are necessary to establish guidelines.

## Introduction

Inflammatory bowel disease (IBD) encompasses the diagnoses of Crohn’s disease and ulcerative colitis, and the prevalence has increased in the United States over the last few decades [1]. Fifty percent of patients are diagnosed before 35 years of age with twenty-five percent of patients conceiving for the first time after diagnosis [2]. For women of childbearing age, there are reproductive consequences from IBD. Active disease at conception increases the risk for worsening disease, with disease flares being more common in patients with ulcerative colitis than Crohn’s disease [3]. For patients who conceive with active disease, 50-70% of patients will have chronic or worsening disease that is more difficult to treat [4-6]. A previous study reviewing the literature concluded that treatment is recommended and safe with certain medications in pregnancy, breastfeeding is encouraged, and more prospective studies are needed about the management of IBD in patients of childbearing age [7].

This case report details the course of a patient with untreated and worsening ulcerative colitis throughout pregnancy who developed a rectovaginal fistula, a morbid and preventable complication.

## Case presentation

A 37-year-old G1 with a body mass index (BMI) of 21 presented to an urban underserved obstetrics clinic with an unplanned and initially undesired pregnancy. Pregnancy dating was established by a nine-week ultrasound that was not consistent with her last menstrual period. As per the patient, she reported medical history notable for Clostridium difficile colitis and an unremarkable surgical and family history. She reported taking a daily probiotic. Social history was remarkable for an Edinburgh Postnatal Depression Score (EPDS) of three and she reported being employed, living alone, and feeling safe at home, although she no longer had contact with the father of the child. The routine prenatal laboratory panel was unremarkable with a hemoglobin of 13.2 and infectious disease workup negative for hepatitis B and C, syphilis, chlamydia, and gonorrhea. Physical exam findings were unremarkable with normal breast, cardiovascular, respiratory, abdomen, and pelvic examinations.

At 22 weeks, the patient reported symptoms of another Clostridium difficile flare with loose bloody stools and multiple daily bowel movements associated with abdominal cramping and nausea. No laboratory tests were ordered by the obstetric provider as she had an appointment with gastroenterology three weeks later that she was trying to move up. After the gastroenterology appointment, the notes became visible to the obstetrics providers. The notes detailed a diagnosis of severe ulcerative pancolitis that was initially diagnosed 14 years ago via colonoscopy, although images and histopathology details were unavailable for the obstetrics providers to review. Throughout the prior 14 years, she had intermittently received treatment with mesalamine although had been noncompliant with treatment and had intermittent lapses in her care. Laboratory studies were remarkable for hemoglobin 10.3, mean corpuscular volume (MCV) 100.3, platelet 340,000, white blood cell (WBC) 7.0, C-reactive protein (CRP) 53, and erythrocyte sedimentation rate (ESR) 25. Despite extensive counseling about the indications and safety of antepartum treatment at the gastroenterology appointment, she refused to restart medications and believed her condition could be managed with a gluten-free diet.

Throughout the third trimester, she reported improvement in her ulcerative colitis symptoms although she continued to have daily bloody stools and developed microcytic iron-deficiency anemia (hemoglobin 9.0, platelet 338,000, WBC 6.59, MCV 95, ferritin 11, folate 20, vitamin B12 585). She was started on oral iron supplementation and received one intravenous iron infusion. A growth ultrasound at 34 weeks confirmed a fetus with appropriate growth at the 49th percentile. At 40 weeks, the patient called the obstetric clinic with concerns about a “natural birth” and whether this would be “right for [her].” Upon further questioning, she reported the worst colitis flare of her life along with leakage of stool from the vagina when she has loose stools, fecal matter mixed with vaginal discharge, and fecal matter on her underwear despite cleaning her anus after bowel movements. She informed the obstetrics provider that her gastroenterology provider did not want to start treatment during her pregnancy, and she had not revealed these obstetrics appointments as she did not think it would help.

She was instructed to present to the obstetrics triage unit where physical exam was notable for normal appearance and soft abdomen with a gravid and non-tender uterus. During a pelvic exam, the anus was noted to be intact and clean, although fecal matter was noted along the vaginal introitus. The vaginal discharge appeared to be speckled with fecal matter. There was no visible fistula during the speculum exam, but digital palpation of the vagina increased the amount of brown matter on the examiner’s glove.

Colorectal surgery was consulted and recommended intraoperative evaluation. No additional laboratory or imaging studies were recommended or performed prior to delivery. The amount of inflammation in her pelvis, along with the risks of potential enteric injury during labor, was thought to outweigh the risks of a planned primary cesarean delivery. Colorectal surgery recommended an intraoperative exam under anesthesia, flexible sigmoidoscopy, and possible biopsy to add minimal operative time and decrease her morbidity. Despite extensive counseling, the patient adamantly refused these additional procedures.

Cesarean delivery was performed in the standard fashion, and the uterus was exteriorized without complication with colorectal surgery on standby. Quantified blood loss was 450 milliliters. Apgar scores were eight and nine at one and five minutes, respectively, and the infant weighed 3.02 kilograms. Vaginal blood mixed with fecal matter extruding from the vagina was noted during postoperative fundal massages.

Her inpatient postoperative course was complicated by anemia (hemoglobin 8.3) for which she remained on supplemental oral iron and received one dose of intravenous iron. EPDS score was one, she breastfed without difficulty, and she planned to use condoms for contraception. At five weeks postpartum, she was concerned about a wound infection and had an acute appointment at which time a normal-appearing incision was noted. She then skipped her routine six-week postpartum appointment and further attempts to schedule follow-up appointments were not returned.

Four months postpartum, she returned to the gastroenterology clinic with complaints of twenty bowel movements per day along with rectal bleeding and worsening abdominal pain. She had significant weight loss down to an underweight BMI of 17.7 which caused her to stop breastfeeding. Laboratory tests were remarkable for a fecal calprotectin over 5000 and ESR 68. At this visit, she was agreeable to starting treatment with mesalamine and prednisolone, and a colonoscopy was scheduled. One month later, she had improved symptomatically and gained weight back to a normal BMI of 19.4. She no-showed her scheduled colonoscopy at six months postpartum and has since been lost to follow-up.

## Discussion

Rectovaginal fistulas are an uncommon and morbid complication of severe ulcerative colitis due to chronic inflammation and poor tissue integrity, with few cases reported in the literature [8]. Poorly controlled ulcerative colitis or non-adherence to medication regimens increases the risk of disease relapse and progression in pregnancy [9]. Although the exact mechanism remains unknown, potential factors include the immunocompromised state of pregnancy, cytokines released from the placenta, and cessation of tobacco use [10]. Disease management during pregnancy is imperative for prevention of adverse maternal or fetal outcomes, and there are multiple medications safe for use in pregnancy [9,11]. Patients with ulcerative pancolitis or worsening disease in pregnancy may be at higher risk for low-birth-weight infants, postpartum hemorrhage, and preterm delivery [11-14]. Furthermore, the delivery method for patients with ulcerative colitis should be individualized pending the severity of their perianal disease [10]. One database study identified that patients with IBD underwent primary cesarean sections more often than the general patient population, and this may not be indicated if there is a lack of perianal disease [15]. The American College of Obstetricians and Gynecologists has detailed recommendations for the diagnosis and management of patients with IBD but lacks specific recommendations regarding delivery planning, which is an area necessitating further research and guidelines [16]. To best care for these patients, obstetricians should advocate for their patients to receive treatment for IBD during prepregnancy counseling and antepartum visits to prevent morbid maternal or adverse pregnancy outcomes [17].

## Conclusions

This case details the progression of poorly controlled ulcerative pancolitis throughout pregnancy resulting in a rectovaginal fistula. For patients hesitant to receive treatment for ulcerative colitis during pregnancy, a multidisciplinary team approach should be utilized to identify barriers to care, prevent disease progression, and optimize pregnancy outcomes.
